# “Interesting and useful, extreme and ultimate”: an interview with Prof. Huigao Duan

**DOI:** 10.1038/s41377-023-01261-9

**Published:** 2023-09-12

**Authors:** Shuai Ding

**Affiliations:** Light Publishing Group, Changchun Institute of Optics, Fine Mechanics and, Physics, Chinese Academy of Sciences, 3888 Dong Nan Hu Road, 130033 Changchun, China

**Keywords:** Micro-optics, Ultrafast photonics

## Abstract

He is an explorer in micro-nano manufacturing, an adventurer in interdisciplinary studies, an impassioned educator; an ever-curious reader, a lover of sports, and a coffee connoisseur. In this episode of Light People Interview, we are honored to have Prof. Huigao Duan from Hunan University share his insights on micro-nano manufacturing, planar optics, teaching, nurturing, reading, growing up, and hobbies. “A man with a great smile”, “a good teacher,” and “an interesting soul” are just some of the descriptions that came through my mind during my conversation with him. Today, let’s get to know this outstanding scholar who pursues “interesting and useful, extreme and ultimate” in micro-nano manufacturing and optics.

## Abstract (web summary)

An outstanding scholar who pursues “interesting and useful, extreme and ultimate” in micro-nano manufacturing and optics.

## Graphical abstract

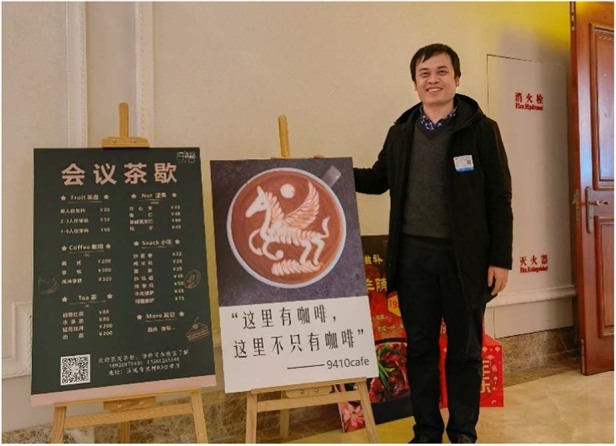


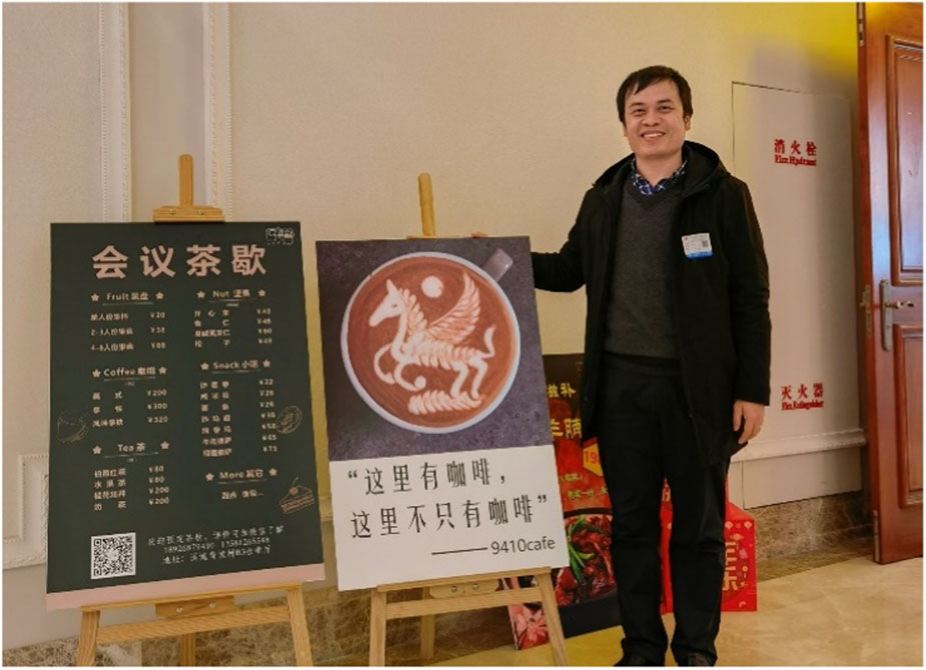


**Short Bio:** Prof. Huigao Duan is a principal investigator in the College of Mechanical and Vehicle Engineering of Hunan University, President of the Greater Bay Area Institute for Innovation of Hunan University, deputy director of the National Engineering Research Center for High-Efficiency Grinding, recipient of the National 100 Outstanding Doctoral Theses and the National Science Fund for Excellent Young Scholars. Having received his BS and Ph.D. in physics from Lanzhou University in 2004 and 2010, he has studied and worked at the Institute of Electrical Engineering, Chinese Academy of Sciences, the Massachusetts Institute of Technology (USA), Institute of Materials Research and Engineering, A*STAR (Singapore), the University of Stuttgart (Germany), and the University of Southampton (UK), etc. He joined Hunan University as a full professor in 2012 and is currently engaged in research on micro-nano manufacturing and micro-system technology. He has authored or co-authored more than 240 papers in domestic and international journals such as *Light: Science & Applications*, *Nature Nanotechnology*, *Nature Energy*, *Nature Communications*, etc. He holds more than 60 invention patents and has presided over more than 10 national projects, including the National Key R&D Program and the National Natural Science Foundation of China. He is the co-Editor-in-Chief of the *International Journal of Extreme Manufacturing*, an editorial board member or associate editor of Research, *IEEE Transactions on Nanotechnology*, *Microelectronic Engineering*, *Optics and Precision Engineering*, etc., and a young correspondence expert of Engineering, the Official Journal of *Chinese Academy of Engineering*.

**Q1. Your main research interests are focused on the field of micro-nano manufacturing. Your team has produced many excellent results in the research of extreme micro-nano manufacturing and micro-systems and published more than 240 SCI papers, including more than 100 IF** **>** **10 papers and 11 ESI highly cited papers, which makes your team the leader in the field. What are the future trends and challenges in the field? What is the main research of your team, and where will the next efforts be focused?**

A1: Today’s information-based society, intelligent products, and accurate medical care have all been made possible by advances in chip technology. From electronic, photonic, optoelectronic, sensing, and biochips to micro-optoelectronic systems, chips have profoundly changed and will continue to change the way of life of human beings, and to a certain extent, redefine the high-end manufacturing industry and even the international political and economic landscape. The pursuit of thinner, lighter, and more-integrated high-performance chip products is the eternal theme of the chip industry. Its core driver is the extremely small-scale, high-precision micro-nano manufacturing technology.

From an academic point of view, compared to electronic chip which is gradually approaching its limits, the development and application of other types of chips, such as photonic chips, quantum chips, biochips, and micro-opto-electromechanical systems in the broad sense are still in their early stages, harboring huge opportunities for innovation. Their development is expected to have a more significant role in promoting social change and human lifestyles, with its most direct output being micro-system technology, which will make all kinds of equipment and systems, instrumentation, and consumer products that can be significantly reduced in size and weight while improving performance and expanding functionality, making them thin, light and small with portability. Again, micro-nano manufacturing is the common enabling technology to achieve these goals.

About future trends and challenges, they vary from application to application, for example, how to balance scale, accuracy, speed, and dimensionality, how to achieve zero defects in the manufacturing of massive micro-nano structures, and how to achieve 3D macroscale manufacturing of atomic and near-atomic scale dot matrix structures. In general, the cross-scale/multi-dimensional macroscale design and manufacturing of small-scale/high-precision functional micro-nano structures for high-performance and extreme service-performance requirements is a common goal and a challenge for most applications. The interplay of function, performance, material, process, and manufacturing cost requires research on integrated design and manufacturing by simultaneously considering the functionality, materials, and processes under the service-performance constraints. These studies often confront the massive data and uncertainties associated with cross-scale, multi-dimensional, multi-material, and multi-processes.

**Figure 1:**
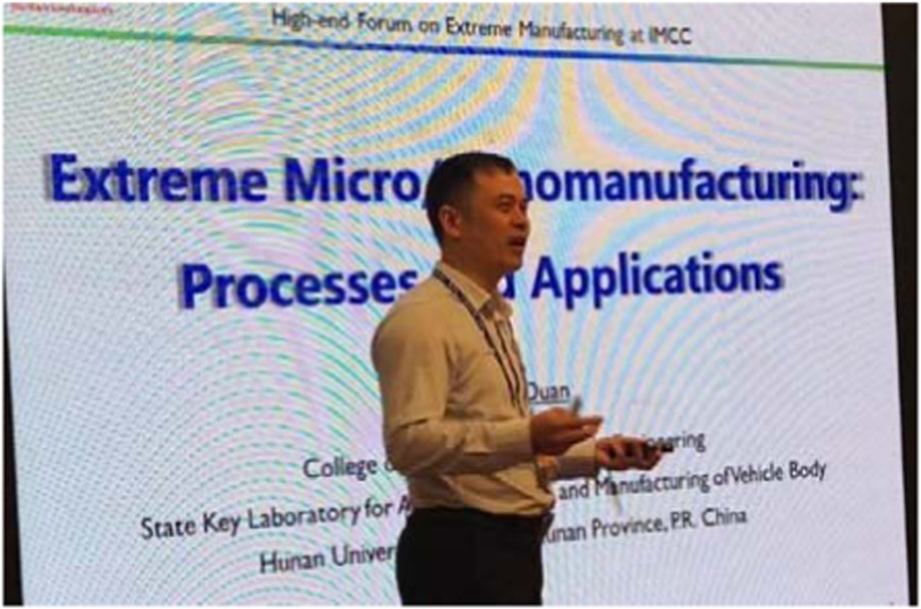
**Prof. Duan giving a presentation at the High-End Forum on Extreme Manufacturing (Shenyang, 2019)**

Our team has been working on three fronts around the above issues. On the one hand, we are promoting the advancement of micro-nano manufacturing technology, trying to pursue manufacturing limits. We mainly use energy beams such as electron beams, ion beams, and laser beams, combined with semiconductor processes such as coating, etching, and material growth while introducing mechanical manufacturing means, such as polishing, forming, and assembly, to develop powerful techniques for the extreme needs of various applications, providing toolboxes, and solutions for manufacturing technology. In the second area, we focus more on fundamental frontier research, mainly on small-scale effects, including both small-scale effects in manufacturing, such as new effects in mechanics, thermodynamics, and dynamics, as well as small-scale effects in structures and devices, such as new effects in electronics, photonics, acoustics, including the interface and quantum effects, to discover new effects and generate new knowledge. The third aspect is to design and fabricate micro-nano devices with unique functions and properties based on the special effects of micro-nano structures, such as micro-optics, micro-sensing, and micro-energy devices, which ultimately serve micro-systems.

Our next step is to continue to promote the development of extremely small-scale and high-precision manufacturing technologies, especially to explore new principles, new processes, and new equipment for manufacturing at the atomic-molecular scale and precision. For example, I have always believed that electron beam/ion beam technology still has a lot of application space to be explored in the field of extreme micro-nano manufacturing, and we have been doing so. On the other hand, our efforts are at the device level, based on our theoretical foundation and practical experience in the field of micro-nano manufacturing. We will focus our research on micro and nano-optical devices and system integration, and expect to make a breakthrough in the research and development of multi-functional, high-performance, ultra-intelligent systems with integrated optical, mechanical, electrical, and computational capabilities for extreme service conditions.

In fact, our team has set the tractive goal of “We Make Things Smaller” since about 10 years ago, hoping to make structures, components, and parts smaller and smaller through innovations in design, materials, and processes, and ultimately to achieve the miniaturization of functional modules and systems.

**Q2. In 2019, your team made important progress in the field of 3D-Integrated metasurfaces for full-color holography: a single integrated color filter microarray based on a stepwise Fabry-Pérot cavity is integrated with a holographic metasurface in a longitudinal stack to achieve low crosstalk, large field of view and polarization-independent full-color holography. The device can be designed to achieve dual display functions of structural color and holography, which makes it promising for a wide range of applications in the fields of holographic display, encryption, etc.** ^1^ **. Three years have passed, has the display technology been applied so far? What are its main advantages and features?**

A2: Yes, in this work, we proposed the concept of 3D-integrated metasurfaces, where the metasurface is vertically integrated with some planar active or passive devices to form an ultra-compact micro-optical system. This work integrates the holographic metasurface with a single integrated FP-cavity color filter array in the vertical direction. The color filter’s low-crosstalk color filtering combined with the metasurface’s phase modulation enables high-quality full-color holography. By decoupling the color information and holography information via the algorithm, the structural colors and holograms can be simultaneously displayed. Based on this work, we collaborated with Prof. Linsen Chen and Prof. Wen Qiao’s group at Soochow University to apply this idea of integrated metasurfaces to naked-eye 3D displays^2^, in which the metagratings and the flat panel display are vertically integrated to achieve a full-color 3D display with spatially variable information density and a horizontal viewing angle of 160° using a flat metagrating to regulate the viewing angle of different shapes. Due to its thin and light profile, the proposed 3D system can be integrated with existing flat panel displays, making it promising for use in portable electronics. In addition, we have recently been conducting research on the integration of metasurfaces and planar detectors and using minimalist metasurfaces to realize angular momentum holography for optical nested encryptions^3^.

**Figure 2:**
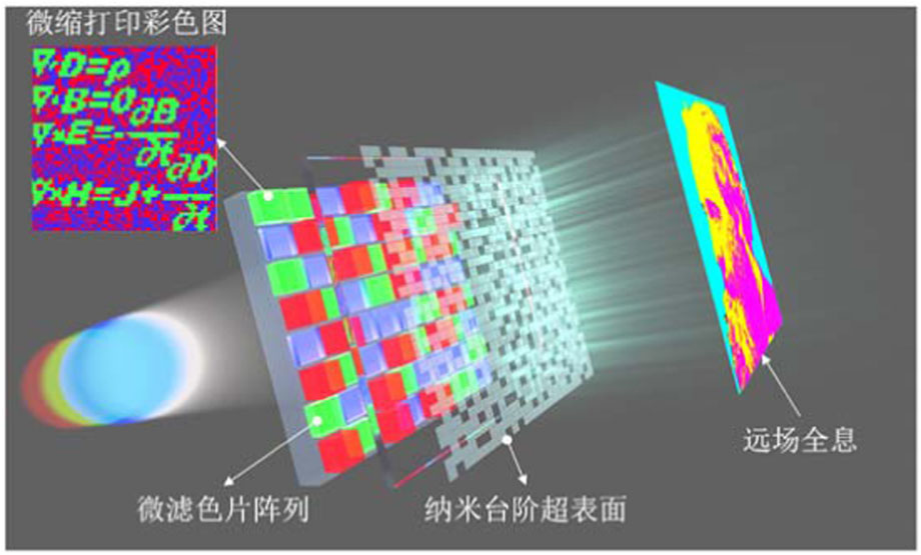
**Schematic diagram of the multi-functional 3D-integrated metasurface**

The 3D-integrated metasurface can take full advantage of the metasurface’s ultra-flat and ultra-thin properties, and the vertical integration of the metasurface with some active or passive devices will significantly improve the system integration. For example, the integration with the emitting devices such as the flat display can realize full-color holographic display and light-field imaging, the integration with modulation elements such as color filters and polarization elements can realize the modulation of multi-dimensional light fields, and the integration with the detecting end of the flat detector can form a multi-dimensional information detector.

**Q3. Last year, you and your team developed a multi-tasking intelligent detection chip** ^4^ **. Two reviewers mentioned that “this is the first experimental implementation of an on-chip diffraction neural network” and that it is an important step towards the practical application of optical neural networks. Where did you get the inspiration for your research? What are the prospects for this technology? What changes will it bring?**

A3: Yes, this is very interesting work that we completed last year. That project is still within the realm of 3D-integrated metasurfaces. After completing the work on integrated metasurfaces in 2019, we have been thinking about how to further exploit the benefits of metasurface integration. We thought about integration with active devices such as lasers on the light emitting side, displays or photodetectors on the detection side. As it happens, in 2018, Prof. Aydogan Ozcan’s team at UCLA, in a *Science* paper, proposed a new diffraction neural network architecture that performs artificial neural network algorithms by training multiple phase diffraction surfaces, which is a 3D optical transmission transformation scheme that enables surface-to-surface parallel computing to perform specific intelligent classification and recognition functions, and it caught our attention. However, the all-optical diffraction neural networks implemented at that time were generally used in non-optical wavelengths such as terahertz and microwave wavelengths, and most devices were relatively large, so we studied the possibility of using a metasurface to bring the system to the optical wavelengths and miniaturize it significantly and proposed the integration of a metasurface all-optical diffraction network and detector in the visible wavelengths. At the same time, we thought about taking advantage of the multiparameter manipulation capability of the metasurface, so we designed a polarization multi-channel metasurface, so that multiple independent tasks could be performed, ultimately forming a new chip-level multi-tasking intelligent sensing architecture so that optical information can be processed directly at the physical layer, which is expected to be used in machine vision, autonomous driving and precision medicine for low-power and fast image processing.

**Figure 3:**
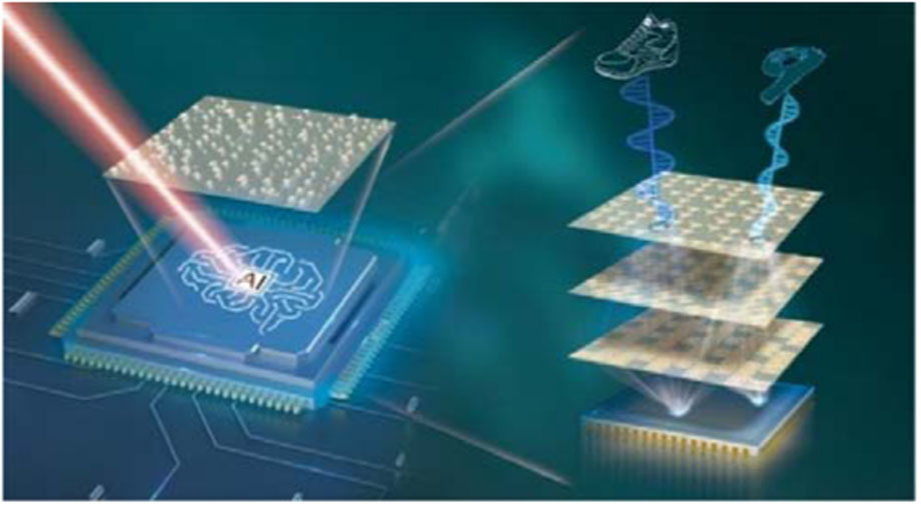
**Schematic diagram of a multi-tasking on-chip diffraction neural network device**^5^

Such a new architecture opens an extensive range of applications in the optical band. As the input signal to the all-optical diffraction network is a two-dimensional matrix, its significant advantage is for image recognition. Therefore, our group believes that light perception-related applications, such as industrial vision, security surveillance, face recognition, and autonomous driving, are good application scenarios. In traditional object recognition applications, the light information of the target object is downscaled to two-dimensional intensity information. After the information is turned into an electrical signal via image sensors, it still needs to be processed by many software or hardware algorithms to obtain the final result. The power consumption and timeliness of the photoelectric conversion in large surface array detectors and the execution of the software and hardware algorithms will be of great concern. Specifically, the architecture has a series of advantages in these applications, such as (1) reduction of power consumption via avoiding a large number of algorithms; (2) computation partially executed at the speed of light, increasing the timeliness; (3) processing of high-resolution images without the need for a large surface array image sensor, reducing the energy and time consumed by the photoelectric conversion of the sensor; (4) metasurface manufacturing is compatible with CMOS processes, promising to be manufactured together with image sensors on a large scale.

These advantages are significant for developing the next generation of photodetectors. However, the current network architecture is linear and relatively simple to perform tasks, and there is still a long way to go before real applications. It is conceivable that in the future, such devices with high-speed on-chip computing may find application in any field where photodetection and recognition are available.

**Q4. With the development of modern production life, the demand for lightweight and miniaturized optical imaging systems has posed new challenges to the development of imaging technology. Metalenses have been one of the hottest research areas in recent years. Among them, fully dielectric metalenses are favored by researchers over metalenses containing metals due to their higher focusing and imaging efficiency** ^6^ **. Your team has recently published a review article summarizing the progress and challenges in this field. Which direction do you think the next developments in metalenses will focus on?**

A4: As one of the most important applications of metasurfaces, metalenses have attracted widespread interest. After the proposal of fully dielectric metalenses in 2016, the efficiency of metalenses has increased significantly. People seemed to see the possibility of replacing refractive lenses with metalenses, and many excellent works followed, such as broadband achromatic imaging, large field-of-view imaging, and multi-information imaging (3D, polarization, spectroscopy). But slowly, it became clear that many bottlenecks behind these works still need to be overcome, such as how to achieve large-area achromatic designs. How to further improve the efficiency of metalenses? How to realize volume manufacturing at a low cost? These challenges exist in the whole process of designing, manufacturing, and applying metalenses and have to be addressed in the development of the metalenses.

In terms of design, the multi-functional integration of metalenses is its greatest strength, so the simplification of optical systems by integrating multiple functions into a single lens will continue to be the development direction. The trend will be to integrate polarization, spectral and 3D information into a single system, thus enabling multi-information imaging and detection systems. In addition, it is extremely challenging to balance the various performance requirements such as efficiency, chromatic aberration, and field of view in the design of single-layer metasurface lenses. New design strategies have also been proposed, such as global inverse design methods, hybrid designs of refractive and metalenses, and combining metalenses with computational imaging to optimize imaging results using back-end algorithms.

In manufacturing, how to fabricate metalenses in a large area and at a low cost has been a concern for the industry. At present, efficient preparation is generally divided into two options, one is to use nanoimprinting for replication, and the other is to process through standard CMOS processes. From the reported results, the standard CMOS process may be more suitable for high-volume manufacturing. Still, it cannot be ruled out that the nanoimprinting technology can also meet the demand for high-volume manufacturing after continuous improvement.

In terms of applications, we have seen this year that some companies have already started to launch metalens products, and some have even been applied to practical scenarios. However, wide applications of metalens are still in its early stage. Therefore, continuously exploring the irreplaceable application scenarios of metalenses is the direction that academia and industry need to work on.

Q5. You have studied at or visited the Massachusetts Institute of Technology (MIT) in the USA, the Agency for Science, Technology and Research (A*STAR) in Singapore, University of Stuttgart in Germany, the Max Planck Institute, and the University of Southampton in the UK, and you have traveled throughout North America, Asia, and Europe. How did you feel in different environments? Do you have any advice for young scholars or students of today about studying abroad?

A5: I have indeed gained a lot. For example, in terms of academics, I have broadened my academic horizons, developed my own unique academic taste, and developed an open, collaborative, and inclusive research style. However, I think the biggest gain is the attitude towards academics, that is, “pursue the best”. As long as it is relevant to academic matters, we have to do our best and be professional. For example, we should understand the principles more deeply than others, be better at using the equipment than others, the papers need to be refined word by word, and PPTs need to be optimized image by image and punctuation by punctuation. Another bonus is how to tell a story in an interesting way. The development of science and technology needs to be widely communicated and effectively disseminated, as well as understood by the public and policymakers, and we need to encourage more younger students to engage in research. The ability to tell a story in an interesting and accessible way is something that a researcher needs to develop.

Although it has been 10 years since I was in different countries and groups, I still remember the academic discussions over a cup of coffee, asking questions, making sketches, challenging each other, debating and thinking until I understood the most basic concepts. There was very little discussion of anything other than academic matters, and the general feeling was that the atmosphere was very enjoyable. I have seen a similar atmosphere in an increasing number of settings in China, such as the café in the M building of the Institute of Physics of the Chinese Academy of Sciences, where I like to hang out when I am in Beijing. In an academic lecture hall at the National University of Defense Technology, I once read a quote that resonated with me, “Stupid questions are not only allowed, they are welcome”, and in such an atmosphere, I think we are getting closer to the truth.

About studying abroad, I always encourage younger students to do so. On the one hand, we should always learn from the best group of scientists. Looking both at home and abroad, it is undoubtedly that there are more options. On the other hand, we should have more exposure to people from different research backgrounds and cultures at a young age to better participate in the global academic community, which will help us address global challenges and develop international collaborations in the future.

**Figure 4:**
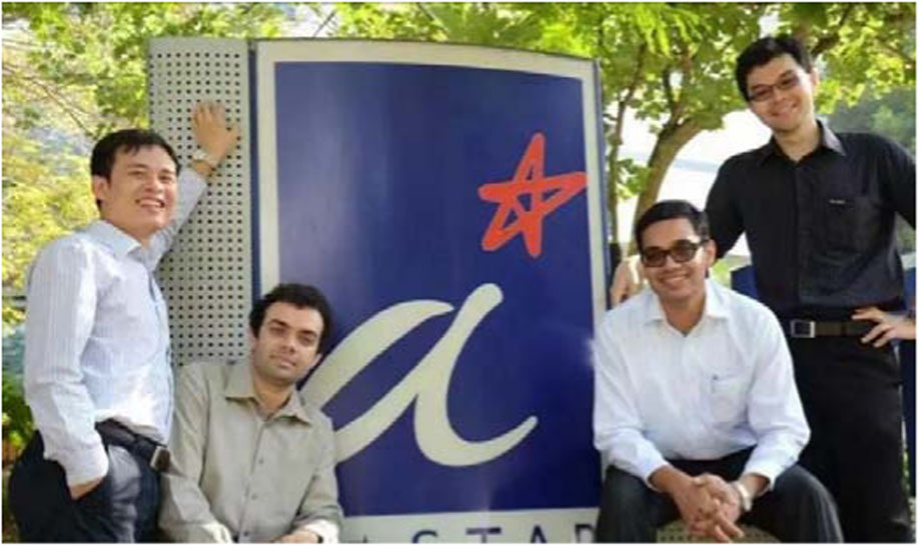
Team photo of Prof. Duan at the Agency for Science, Technology and Research (A*STAR, Singapore, 2012)

**Q6. In your career, you moved from the School of Physics to the School of Mechanics, combining backgrounds in both science and engineering. Do you think this interdisciplinary experience has positively impacted your research? Can you share with us some of the interesting stories?**

A6: I studied physics for my undergraduate degree and chose a sub-discipline in semiconductor physics and devices in my senior year. Whether in the School of Physics or the School of Mechanics, the main line of my research has not changed but always revolves around extreme micro-nano manufacturing and its related applications. When I was in the School of Physics, we mainly aimed at small-scale effects, which were more relevant to basic research, and tended to answer scientific questions to push the boundaries of knowledge. In the School of Mechanics, we take high-performance equipment as the traction, which is more relevant to application-oriented research and focuses on how to make components or parts that could meet the demand of equipment applications.

**Figure 5:**
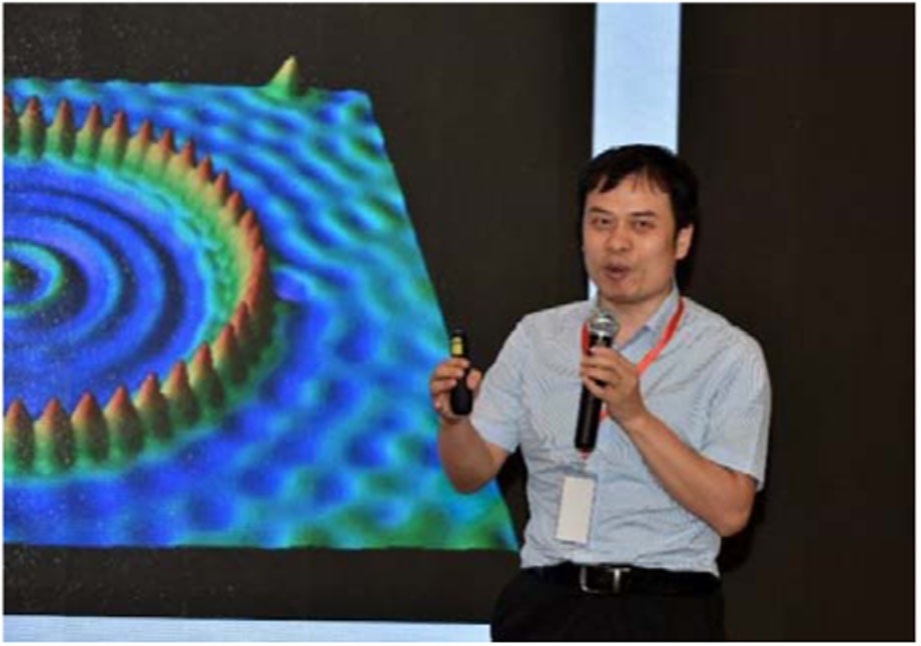
**Prof. Duan sharing insights on “Atomic Scale and Precision Manufacturing, Quantum Functional and Performance Devices**”

In terms of the positive impact on research, this dual background has enabled me to think scientifically as well as practically, and to develop a “Solving the Science Problems via an Engineer’s Thinking Model, Addressing the Engineering Issues via a scientist’s Thinking Model” mindset in solving practical scientific or engineering problems. For experimental disciplines, in scientific research, answering scientific questions often requires sophisticated design, processing, and testing techniques, and thinking like an engineer can help us to better plan experiments, optimize processes and reduce the cycle times. On the other hand, when addressing engineering issues, finding the key scientific problem behind the engineering issues is extremely helpful.

As an anecdote, I often laugh at myself as a physicist who knows a bit of mechanics and a mechanical engineer who knows a bit of physics, but there is one thing in common: optics. Optics is a very good link between physics and mechanical engineering, just like the relationship between photophysics and optical precision mechanics. From an application point of view, extreme manufacturing and extreme optics can empower and support each other. The interplay between the two is even more evident at the micro and nanoscale, especially at the atomic-molecular scale.

**Q7. Jorge Luis Borges (Argentina) once said, “I have always imagined that Paradise will be a kind of library.” I understand that you also presided over the library of Hunan University, during which time you were active in organizing various communication activities, developing library surroundings and creative ideas, and giving a presentation on “Reading, Growing and Libraries: My Experience on Personal Reading”. Can you tell us about your relationship with the library and the different experiences you have had in your various positions (professor, director, dean, etc.), and your experience of the way you work?**

A7: I am not so much a librarian as I am a professor who wants to get more people to come to the library and learn how to read. A university professor is more of an educator. As an educator, I have always believed that the environment and atmosphere of public spaces such as library, gymnasium or café plays a crucial role in implicit education, probably because I myself am a believer of the “atmosphere group”. During my time as a librarian, we organized various activities to create a high-quality second classroom where the atmosphere, environment, and resources are attractive to students by spending their time reading and improving their information literacy.

I particularly like to visit bookstores, buy books, and visit libraries. Since my university days, I have spent most of my self-study time in the library, and I really enjoy the atmosphere there. I enjoy the atmosphere in the library, walking through the shelves, choosing several books on a topic and reading them in comparison, sometimes puzzling over them, sometimes scratching my head, sometimes reading ten pages a second, ten lines at a time. I enjoy the lifestyle of spending a whole afternoon in the library, often with great mental satisfaction while solving puzzles. In addition to books related to scientific research, books on the social sciences and humanities are also very important, especially for educators. We often discuss the importance of “intelligence, emotional intelligence and adversity”, but one’s “beauty, pleasure and fun” quotient is also significant. Undoubtedly, reading “useless books” that are not directly related to one’s subject is crucial.

Regarding different roles, no matter how many roles I hold, the professor is still the most basic role. The role of a librarian was more about planning and organizing reading and information services for students and faculties, while the role of a dean is more about exploring and practicing the in-depth integration of industry, university, and research. These roles complement each other and are an organic whole. From the perspective of personal growth, taking up different roles, if one is truly committed, one can achieve “learning via doing”, thus making one’s understanding of education, research, and innovation deeper and making oneself a better educator, which will, of course, be conducive to cultivating better and more complete talents.

In terms of working methods and approaches, as one’s tasks gradually increase, one has to make some trade-offs to balance human resources training, scientific research, and public service, and teamwork is also very important. For example, for scientific research, we need to gradually focus on the topics that we are best at, most interested in, and of the greatest importance, and we need to strengthen cooperation with researchers who have complementary strengths. I have briefly summarized the five work methodologies, which may not be suitable to everyone but are easy to remember, namely Hardwork, Teamwork, Network, Framework and at the bottom, Homework. When doing research, we should work hard with a team and more collaborators and eventually should develop our own research framework that is relatively stable for years. Of course, all of these require support and understanding from the family.

**Q8. You have a lot of experience in teaching. What do you think is the most important aspect of teaching and educating people? What qualities are needed for postgraduate students to do well in academic research?**

A8: I still have much room for improvement in educating students and don’t have many skills, but I think one thing is most important in teaching and educating people: “learning to be a teacher and being a model”. Whether being a person, performing a task, or being an academic, we have to set high standards for ourselves so that we can be a role model for our students. In addition, as a teacher, we must be willing to serve as a ladder and do our best to provide the best resources, create more opportunities and create a relaxed and inclusive atmosphere for our students.

For postgraduate students engaged in experimental science, a solid theoretical foundation, an open academic vision, a rigorous academic attitude, a lot of hands-on practice, and good communication, expression, and writing skills are the basis for doing good academic research. I also often ask myself to use the 4 optimizations: do the most meaningful topic with limited time, maximize the output with limited input, give the optimal solution in the shortest time, and express the deepest meaning with the fewest words. These can indeed evaluate one’s basic skills, which should be trained in a targeted manner during the student days. The phrase “when you are at the top of the mountain, you will see all the mountains” provides us with a good methodology for doing research, although different people may have different understandings of it.

If we summarize the previous questions about study abroad, disciplines, and jobs, for students and ourselves, “multi-culturalism, multi-disciplinarity and multi-job training” is helpful at different stages of growth, either in a direct or indirect way.

**Q9. You have put a lot of effort into building and developing the journal** ***International Journal of Extreme Manufacturing*, and it has achieved remarkable results with its current impact factor of 14.7. You have been active at the forefront of journal building, and you are also a contributing author of the Light group of journals. Please tell us about your expectations and vision for the Light Journal group?**

A9: Academic journals are a platform for communication among the academic community and a carrier of scientific research and academic achievements. A good journal needs to assume the responsibility of disseminating excellent results and promoting academic development and playing the role of shaping the academic frontier and leading the direction. To run a good journal, it needs an excellent group of authors, readers, and reviewers, as well as a unique and tasteful editorial board and a highly professional editorial team, which often also requires sentiment and careerism.

The Light brand series journals are doing very well. The 21st century is the century of optics, and the Light Publishing Group (LPG) will have great potential. With the joint efforts of all of us, the “Light” brand will become more deeply rooted, and the top journals in the field of optics will emerge. I think the LPG will be successful if the first thing that comes to mind when people have the highest level of results in the field of optics is the Light series journals. Of course, it would also be beneficial for authors and readers if high-level results at different levels and in different directions could always find a home in the Light brand.

**Q10. You have an enviable family with two lovely children, how do you balance family life and work when you are mainly working in Guangzhou and your family is still in Changsha?**

A10: It is indeed challenging to balance. In most cases, I have to take work a priority, I can only ask my family to understand. There is definitely a lot of guilt and regret, such as missing out the quality time with the children and many wonderful moments in their growth. When work allows, I always try to go back to Changsha on weekends to spend time with the children. Actually, even when I am in Changsha, I have an endless list of things to do. However, as long as the family is together, even for a moment, it is joyful.

**Q11. You also love sports in your spare time: volleyball, badminton, etc. Can you tell us about the importance of developing hobbies? What are the positive effects of sports on you?**

A11: We all need to develop our bodies and minds and become social beings. In sports, we can build a healthy body, develop resilience, improve cooperation, and gain comfort from teammates. In group activities, we need to deal with people, we have people to learn from, we can learn from each other, and it is easier to develop empathy ability. A hobby allows us to interact with the world, with others, and with our true selves, in addition to our studies and work, which contributes to our physical and mental health, holistic development, and self-happiness.

**Figure 6:**
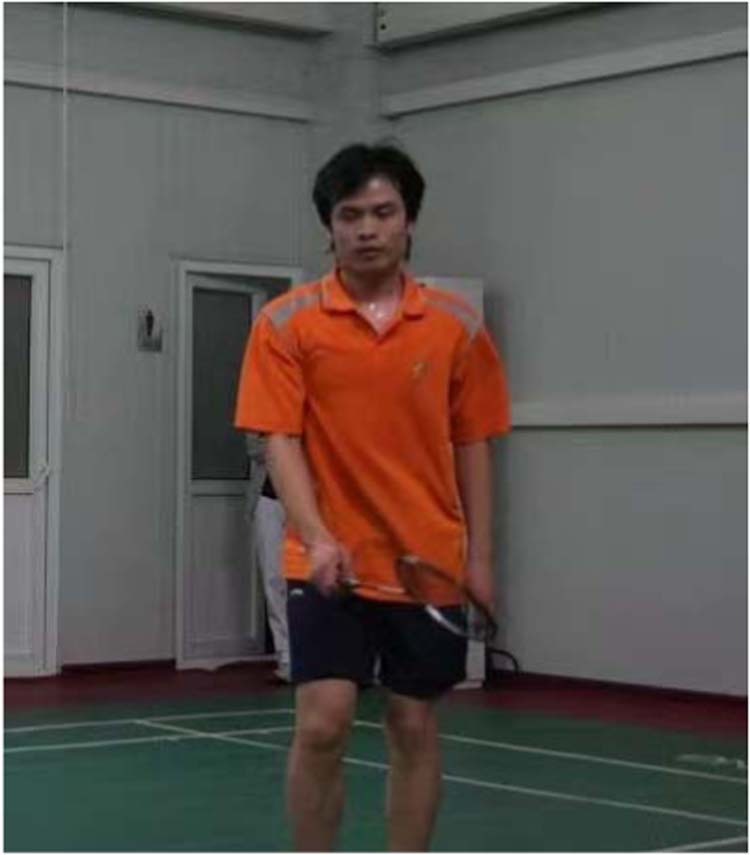
**Prof. Duan participated in a badminton tournament at the Institute of Electrical Engineering, Chinese Academy of Sciences (Beijing, 2008) in his postgraduate student days**

I enjoyed the physical and mental benefits of sweating profusely and being exhausted from exercise when I was young. Now how to lose weight is the main driving force. From a practical point of view, when I’m particularly stressed or anxious and can’t move forward with my work, doing sports makes me more productive and creative. For many brainiacs, exercise is probably the best way to take a break. Unfortunately, with the increasing number of commitments and the changing work environment, the ball games I used to play for years are now a luxury for me, and with it comes weight gain.

In conclusion, I often think it would be nice to read in the library, play sports in the gym and meet friends in cafes, where there is both academia and life, on top of the busy work schedule. Without the epidemic, offline academic activities have restarted. I really look forward to reuniting with old friends and meeting younger new friends. I believe meetings, exchanges and discussions will lead to more sparks. I also welcome all friends to collaborate more through the Light platform!
